# Supplementary material for: Aromatic Characteristics of Passion Fruit Wines Measured by E-Nose, GC-Quadrupole MS, GC-Orbitrap-MS and Sensory Evaluation
Source: Foods. 2022 Nov 24;11(23):3789. doi: 10.3390/foods11233789 (PMC9735701; doi:10.3390/foods11233789)
Supplement: Supplementary file 1 [file foods-11-03789-s001.zip › foods-2018833-supplementary.pdf]

Table S1. The standard curve, validation range, LOD, LOQ, HRF score and coefficient of determination (r2) for sulfur volatile compounds in passion fruit wine.

Standard	HRF score	Formula	M^{+a}	Mass error (ppm)	Calibrated range (μg/L)	r^2	Slope	Intercept	LOD (μg/L)	LOQ
Benzothiazole	99.628	$C_7H_5NS^+$	**135.0137***	0.7021	0.01-43.75	0.9941	217.69	-0.1519	0.0016	0.0053
		$C_6H_4S^+$	108.0029	0.7931						
		$C_4H_2S^+$	81.9872	0.9022						
Diethyl disulfide	97.0433	$C_4H_{10}S_2^+$	**122.0218***	0.2315	0.08-41.00	0.9957	45.71	0.1037	0.0068	0.0204
		$C_2H_6S_2^+$	93.9906	0.6783						
		$CH_3S_2^+$	78.9672	0.7022						
3-(Methylthio)propanoic acid ethyl ester	94.9905	$C_6H_{12}O_2S^+$	**148.0553**	0.1444	0.016-130.00	0.9964	396.81	0.0611	0.0039	0.0129
		$C_3H_6S^+$	74.0185*	0.2424						
		$C_3H_7S^+$	75.0263	0.2631						
3-(Methylthio)propyl acetate	95.1033	$C_6H_{12}O_2S^+$	**148.0553**	0.6668	0.88-113.20	0.9955	3069.50	-6.7311	0.0068	0.0204
		$C_4H_8S^+$	88.0341*	0.3762						
		$C_3H_5O_2^+$	73.0285	0.8079						
Methionol	97.0737	$C_4H_{10}OS^+$	**106.0447***	0.5426	5.16-1320.00	0.9977	17319.00	-32.435	0.0128	0.0428
		$C_4H_8S^+$	88.0342	0.8896						
		$C_3H_5S^+$	73.0107	0.8977						
3-Mercaptohexyl acetate	99.7294	$C_4H_7S^+$	87.0263*	0.2711	0.36-179.04	0.9993	910.40	-1.6925	0.0024	0.0072
		$C_5H_9S^+$	101.0419	0.3188						
		$C_6H_{12}S^+$	116.0655	0.4352						
2-Methyltetrahydrothiophen-3-one	100	$C_5H_80S^+$	**116.0291***	0.4785	0.72-46.38	0.9993	1043.00	-1.2723	0.0026	0.0086
		$C_4H_8S^+$	88.0342	0.5219						
		$C_2H_4S^+$	60.0029	0.7119						
3-Mercaptohexanol	93.3314	$C_6H_{14}OS^+$	**134.0761**	0.6841	0.24-30.88	0.9995	1761.80	-0.0445	0.0467	0.1556
		$C_6H_{10}^+$	82.0777*	0.9026						
		$C_5H_7^+$	67.0543	0.9502						
S-Ethyl ethanethioate	96.0453	$C_4H_80S^+$	**104.0291**	0.5094	1.60-226.00	0.9984	489.79	0.792	0.0128	0.0384
		$C_2H_30^+$	43.0178*	0.4076						
		$C_2H_4S^+$	60.0028	0.8044						

a HRF (High-Resolution Filtering score); percentage of the spectrum obtained by MS Orbitrap that can be explained by combination of accurate mass, library matching and percentage of explained ions observed.
b Exact mass spectra
c Limit of detection
d Limit of quantification
* Quantitative ion
bold: Molecular ion peak

Table S2. Significant differences of OAV among yeast strain ES488 (ES), BV818 (BV), VIC (VI), and CY3079 (CY).

Odor series	ES	BV	VI	CY
Fruity	1591.78±7.07a	288.68±4.24d	330.57±3.19c	372.9±1.98b
Floral	9017.49±7.78b	7342.91±5.23d	8533.87±6.16c	10333.03±7.44a
Herbaceous (or vegetal)	781.72±21.21a	476.5±3.94b	305.76±3.22c	21.8±1.21d
Caramel	228.24±5.66b	180.04±3.46c	252.07±2.83a	253.25±2.23a
Earthy	2.15±0.09a	1.83±0.08a	2.19±0.13a	2.2±0.14a
Chemical	112.05±1.38c	176.81±2.12a	151.64±1.09b	69.6±1.71d
Fatty	280.57±4.93c	321.65±2.79b	355.7±2.55a	366.22±3.62a

Data are the mean ± standard deviation of duplicate tests. Different letters in each row indicate significant difference at a significant level of 0.05.

Table S3. Mean, standard deviation value and Tukey's test of sensory evaluation.

Attribute	ES	BV	VI	CY
Passion fruit	89.44±0.39a	87.93±0.47a	74.1±0.71b	75±0.71b
Mango	47.38±0.44b	49.92±0.37b	58.62±0.48a	60.13±0.61a
Green apple	45.28±0.51b	32.57±0.3d	39.77±0.87c	50.45±0.39a
Lemon	46.54±0.54b	53.56±0.52a	37.94±0.25c	48.07±0.3b
Floral	1.95±0.04d	6.32±0.06c	12.88±0.08b	37.45±0.46a

Data are the mean ± standard deviation of duplicate tests. Different letters in each row indicate significant difference at a significant level of 0.05.

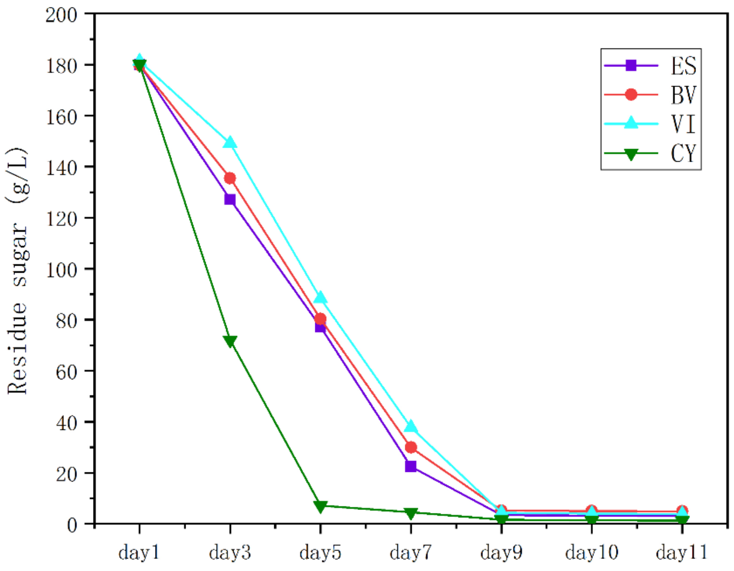

Figure S1. Residual sugar of passion fruit wine fermented by commercial yeast strain ES488 (ES), BV818 (BV), VIC (VI), and CY3079 (CY).
